# A Tri-Stimuli Responsive (Maghemite/PLGA)/Chitosan Nanostructure with Promising Applications in Lung Cancer

**DOI:** 10.3390/pharmaceutics13081232

**Published:** 2021-08-10

**Authors:** Fátima Fernández-Álvarez, Gracia García-García, José L. Arias

**Affiliations:** 1 Department of Pharmacy and Pharmaceutical Technology, Faculty of Pharmacy, University of Granada, 18071 Granada, Spain; fatimaferal@ugr.es; 2 Faculty of Experimental Sciences, Universidad Francisco de Vitoria, 28223 Madrid, Spain; gracia.garcia@ufv.es; 3 Institute of Biopathology and Regenerative Medicine (IBIMER), Center of Biomedical Research (CIBM), University of Granada, 18071 Granada, Spain; 4 Biosanitary Research Institute of Granada (ibs.GRANADA), Andalusian Health Service (SAS), University of Granada, 18071 Granada, Spain

**Keywords:** chitosan, heat-triggered drug release, magnetic drug delivery, pH-responsive drug release, PLGA, triple stimuli-responsive nanoparticle

## Abstract

A (core/shell)/shell nanostructure (production performance ≈ 50%, mean diameter ≈ 330 nm) was built using maghemite, PLGA, and chitosan. An extensive characterization proved the complete inclusion of the maghemite nuclei into the PLGA matrix (by nanoprecipitation solvent evaporation) and the disposition of the chitosan shell onto the nanocomposite (by coacervation). Short-term stability and the adequate magnetism of the nanocomposites were demonstrated by size and electrokinetic determinations, and by defining the first magnetization curve and the responsiveness of the colloid to a permanent magnet, respectively. Safety of the nanoparticles was postulated when considering the results from blood compatibility studies, and toxicity assays against human colonic CCD-18 fibroblasts and colon carcinoma T-84 cells. Cisplatin incorporation to the PLGA matrix generated appropriate loading values (≈15%), and a dual pH- and heat (hyperthermia)-responsive drug release behaviour (≈4.7-fold faster release at pH 5.0 and 45 °C compared to pH 7.4 and 37 °C). The half maximal inhibitory concentration of the cisplatin-loaded nanoparticles against human lung adenocarcinoma A-549 cells was ≈1.6-fold less than that of the free chemotherapeutic. Such a biocompatible and tri-stimuli responsive (maghemite/PLGA)/chitosan nanostructure may found a promising use for the effective treatment of lung cancer.

## 1. Introduction

Control of the in-vivo fate of antitumour agents (genes and/or drugs), when they are site-specific delivered by biodegradable and biocompatible nanoparticles (NPs), has been reported to optimize cancer therapy outcomes in terms of efficacy and safety [1,2,3]. Remarkably, Nanotechnology has also contributed to the implementation of combination therapies against this malignant disease, e.g., chemotherapy and photodynamic therapy [4], chemotherapy and photothermal therapy [5], and chemotherapy and hyperthermia [6].

To these objectives, the introduction of passive and active drug targeting strategies in NP engineering has resulted to be a key aspect to be seriously considered [7,8]. These wise formulation strategics are focused on the surface functionalization of the NP structure with: (i) hydrophilic polymer chains, classically poly(ethylene glycol) (PEG), to develop stealth particles that can exploit the enhanced permeability and retention (EPR) effect commonly found at the tumour site [9]; and, (ii) molecules capable of interacting with receptors at the cancer cell membrane (ligand-mediated targeting) [10]. Also, the nanostructure can be optimized to assure the triggered (selective) release of the chemotherapeutics in deep contact with the malignant cells. The later are stimuli-sensitive drug delivery systems releasing loaded contents on demand when they suffer modifications in their properties and architecture under specific bioenvironmental changes [11]. In this line, the development of nanoparticulate systems exhibiting a pH-responsive drug release behaviour [12,13], or heat (hyperthermia)-triggered drug release capacities [14,15], has been described to facilitate a better accumulation of the chemotherapeutics at the tumour mass. Probably, a more relevant approach to beat the challenges is the formulation of multi-stimuli responsive nanostructures if optimized levels of the antitumour agent are intended in deep contact with the cancer cells or even intracellularly. To that aim, nanoengineering strategies became more complex and difficult to develop [16,17].

In this scenery, superparamagnetic iron oxides are considered to be relevant nanomaterials when engineering the structure of a multi-stimuli sensitive nanomedicine against cancer [18,19,20,21]. Maghemite (*γ*-Fe_2_O_3_) and magnetite (Fe_3_O_4_) are the iron oxide particles preferably introduced in the structure of magnetic nanomedicines [22]. More concretely, including *γ*-Fe_2_O_3_ nuclei in the architecture of nanoparticulate system has been described to report interesting drug delivery properties (efficient tumour targeting in response to a magnetic field located at the cancer site) [23,24,25], magnetic hyperthermia characteristics to trigger drug release and/or to generate a complementary antitumour mechanism [26,27], and magnetic resonance imaging (MRI) contrast properties to visualize the malignant tissue and cells [28,29].

However, the architecture of the iron oxide-based nanosystems must be improved to assure the safety, biocompatibility, and stability of the magnetic colloid, and the vehiculization and targeting of the therapeutic agents (drugs and/or genes). For instance, which is possible when using polymers in the surface functionalization of these NPs: poly(D,L-lactide-*co*-glycolide) (PLGA) [30], chitosan (CS) [31], or poly(*ε*-caprolactone) [32], to cite just three representative examples.

PLGA nanomatrices can be advantageously used in the formulation of magnetic nanocomposites to obtain appropriate loadings and controlled (biphasic) release profiles of chemotherapeutics [33,34,35]. In addition, pH-triggered drug delivery capabilities have been described for this biocompatible copolymer. A consequence of its improved degradation at the acidic intratumoural pH by hydrolysis of the backbone ester linkages in its chemical structure [36,37]. PLGA has been also used to prepare thermoresponsive nanocarriers generating a heat-triggered release of the cargo at the targeted site [38,39]. In fact, it has been defined an increase in the rate of polymer degradation with increasing incubation temperatures [40], and the enhancement of the mobility of the PLGA chains at temperatures over the glass transition temperature (*T*_g_) of PLGA (≈40 °C) [39], being these key factors activating a rapid release of the cargo. Thus, drug release from PLGA matrices could be triggered when superparamagnetic iron oxide nuclei are embedded into them: a high frequency alternating electromagnetic field will transform these magnetic cores into heaters producing an increase in temperature to ≈45 °C [18,22,41], over the *T*_g_ of the polymer. As a result, a fast drug release would take place at this temperature given the greater polymer degradation and also because of the improved drug diffusion of the chemotherapeutic molecules through the PLGA structure.

Like PLGA nanostructures, CS-based nanosystems are efficient in the tumour pH-responsive release of drugs, given the high solubility of the polymer at these acidic pH values [42,43]. Additionally, CS is a biocompatible and water-soluble polymer that could be considered as an alternative to poly(ethylene glycol) (PEG) chains when engineering long-circulating NPs. Hence, surface functionalization with CS may optimize the biodistribution and therapeutic effects of PLGA-based nanomedicines by: (i) providing hydrophilic and positively charged stealth coatings that could reduce or even inhibit protein corona formation, thus minimizing and delaying the opsonization process to evade phagocytosis [44,45,46,47], while favouring the uptake by targeted cells [48,49]; and, (ii) creating an additional barrier to drug diffusion during the early-time burst release of the biphasic drug release profile [50,51]. Incorporation of the CS shell onto a PLGA-based NP may take place by an attractive interaction between the negative PLGA matrix and the positive polysaccharide [52], and the idea has been applied to the production of magnetopolymer particles, i.e., (Fe_3_O_4_/PLGA)/CS [53,54] and (*γ*-Fe_2_O_3_/PLGA)/CS [55] nanostructures. However, the multi-stimuli-responsive character of these (core/shell)/shell composites has not yet been characterized.

Cisplatin, Cisplatinum, or cis-diamminedichloroplatinum(II) (CDDP) dichloride [molecular weight, M_W_: 300.05 g/mol; water solubility: 2.53 mg/mL; *n*-octanol-water partition coefficient (log *P*_OW_): −2.19] has demonstrated an appropriate activity against solid tumours, e.g., lung cancer, ovarian cancer, head and neck squamous cell carcinoma breast cancer, and brain cancer [56], when administered as a single antitumour agent or in a combination chemotherapy regimen. Unfortunately, rapid biodegradation of CDDP molecules in blood could be considered a key limitation to conventional chemotherapy (human plasma half-life ≤ 0.5 h) [56,57]. To beat the challenge, it has been proposed the incorporation of this chemotherapeutic into nanocarriers, e.g., PLGA- [58,59] or CS-based NPs [31,60]. That association has reported optimized pharmacokinetics along with improved contact of the anticancer molecules with the malignant cells. Nevertheless, greater benefits have been associated to the engineering of stimuli-responsive nanostructures for pH-triggered [37,61] and/or heat (hyperthermia)-triggered [41,62] drug release: CDDP release in blood is kept to a minimum while a rapid release is then activated when the pH-responsive colloid reaches the tumour interstitium (pH ≈ 5), and/or when this targeted site is coupled to a high frequency alternating electromagnetic field transforming the superparamagnetic iron oxide cores included in the nanostructure into heaters that produce heat in a controlled manner (maximum temperature of ≈45 °C, not causing damage to healthy tissues surrounding the tumour mass). CDDP has also been previously loaded to non-magnetic PLGA/CS-based particles [49,63], but up to now the colloidal stability, hemocompatibility, and suitability of these (core/shell) nanoparticulate systems for (dual) pH-triggered and heat (hyperthermia)-triggered CDDP release was not characterized.

In this contribution, we have investigated a procedure to formulate NPs in which *γ*-Fe_2_O_3_ nanocores are included into a PLGA matrix loaded with CDDP molecules and surface functionalized by CS. Reproducible production of this (*γ*-Fe_2_O_3_/PLGA)/CS nanostructure was demonstrated by comparing some relevant physical and chemical properties to those of *γ*-Fe_2_O_3_/PLGA and CS particles. Evaluation of the toxicity of the nanocomposites was based on hemocompatibility studies, and tests on human colonic CCD-18 fibroblasts and human colon carcinoma T-84 cells. After defining the short-term stability of the colloid and the in-vitro interaction with BSA, the characterization of the multi-stimuli-responsive capabilities of these (core/shell)/shell composites started with the analysis of their magnetic responsiveness. Then, CDDP loading to the NPs and in-vitro release was evaluated by ultraviolet (UV) spectrophotometry. The potential use of the nanoplatform for (dual) pH-triggered and heat (hyperthermia)-triggered CDDP release was investigated at the acidic microenvironment typical of tumours (pH ≈ 5) and at the temperature used in magnetic hyperthermia to trigger drug release (≈45 °C). To finish, cytotoxicity against human lung adenocarcinoma A-549 cells of these CDDP-loaded magnetic nanocomposites was evaluated. To our best knowledge, this is the first time that these (*γ*-Fe_2_O_3_/PLGA)/CS (core/shell)/shell NPs have demonstrated adequate properties as magnetic-, pH- and temperature-responsive nanostructures for the delivery of CDDP molecules to malignant cells, and promising activity against lung cancer.

## 2. Materials and Methods

### 2.1. Materials

Deionized and filtered water was used in the experiments (Milli-Q Academic^®^, Millipore, Molsheim, France). Resomer^®^ RG 502 H [PLGA, 50:50 poly(D,L-lactide):poly(glycolide), M_W_ ≈ 7 to 17 kDa, inherent viscosity ≈ 0.16 to 0.24 dL/g), low M_W_ CS (≈50 to 190 kDa, determined by viscosity measurement; polydispersity not determined by the laboratory; 75–85% deacetylated; 99% purity level), ethylenediaminotetraacetic acid (EDTA), monosodium citrate [NaH_2_(C_3_H_5_O (COO)_3_)], *cis*-diamminedichloroplatinum(II) (CDDP) dichloride, 3-(4,5-dimethylthiazol-2-yl)-3,5-diphenyl tetrazolium bromide (MTT) solution, Dulbecco’s modified eagle’s medium (DMEM), phosphate buffered saline (PBS), bovine serum albumin (BSA; heat shock fraction, ≥98% purity level), fetal bovine serum (FBS), and Penicillin-Streptomycin solution (containing 10,000 U/mL of Penicillin and 10 mg/mL of Streptomycin) from Merck KGaA (Gernsheim, Germany). Kolliphor^®^ P-188 from BASF (Ludwigshafen, Germany). Iron(III) nitrate nanohydrate [Fe(NO_3_)_3_·9H_2_O, M_W_: 404 g/mol], iron(II) chloride tetrahydrate (FeCl_2_·4H_2_O, M_W_: 198.81 g/mol), potassium nitrate (KNO_3_), perchloric acid (HClO_4_, 70%, American Chemical Society, ACS, specification, Washington, DC, USA), hydrochloric acid (HCl, 37%, ACS specification), ethanol (EtOH, 96°), dimethyl sulfoxide (DMSO), and acetic acid (CH_3_COOH, ≥98%, ACS specification) from VWR lnternational Eurolab S.L.U. (Barcelona, Spain). Iron trichloride hexahydrate (Cl_3_FeH_12_O_6_, M_W_: 270.32 g/mol), polyvinyl alcohol (PVA, M_W_: 72,000 g/mol), HPLC-grade acetone, citric acid (C_6_H_8_O_7_), ammonia (NH_3_, 30%, ACS specification), and sodium hydroxide (NaOH) from Panreac Química S.L.U. (Barcelona, Spain). Disodium phosphate (Na_2_HPO_4_), dipotassium phosphate (K_2_HPO_4_), sodium sulfate (Na_2_SO_4_), sodium chloride (NaCl), potassium chloride (KCl), and oleic acid (≥99%, ACS specification) from Guinama S.L.U. (Valencia, Spain). These chemicals were of analytical quality and used as received without further purification.

### 2.2. Preparation of the (γ-Fe_2_O_3_/PLGA)/CS Nanostructure

**Figure 1:**
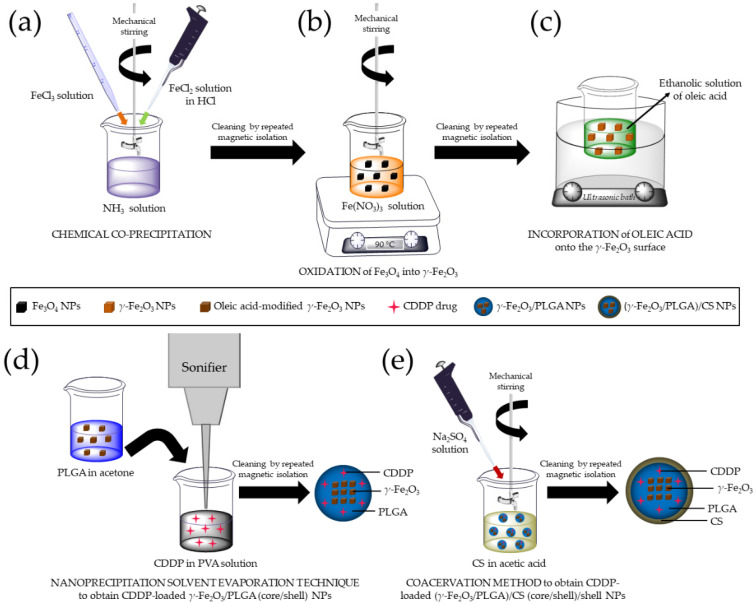
(**a**) Preparation of Fe_3_O_4_ NPs by chemical co-precipitation and (**b**) oxidation into *γ*-Fe_2_O_3_ particles; (**c**) surface modification of *γ*-Fe_2_O_3_ NPs with oleic acid; (**d**) formulation of CDDP-loaded *γ*-Fe_2_O_3_/PLGA (core/shell) particles by nanoprecipitation solvent evaporation; and (**e**) preparation of CDDP-loaded (*γ*-Fe_2_O_3_/PLGA)/CS (core/shell)/shell NPs by coacervation.

The procedure followed to obtain *γ*-Fe_2_O_3_ NPs started with the production of Fe_3_O_4_ particles by chemical co-precipitation (Figure 1a) [64] and finalized with their oxidation into *γ*-Fe_2_O_3_ (Figure 1b) [65] (*n* = 3). To favour the entrapment of the *γ*-Fe_2_O_3_ nanocores into the PLGA matrix, their hydrophilic surface was turned into hydrophobic by using oleic acid [53,66] (Figure 1c) (*n* = 3).

Preparation of the *γ*-Fe_2_O_3_/PLGA NPs was based on the nanoprecipitation solvent evaporation procedure (Figure 1d) [55] (*n* = 3), which has been described to generate PLGA NPs [67]. CDDP loading to the core/shell NPs was accomplished by dissolving the antitumour molecule in 10 mL of the aqueous phase [PVA (1%, *w/v*)], at a given amount (up to 300 μg/mL), before incorporation of 5 mL of the acetone phase (*n* = 3).

Finally, (*γ*-Fe_2_O_3_/PLGA)/CS nanocomposites were formulated by coacervation (Figure 1e) [55] (*n* = 3), a method habitually used to prepare CS NPs [68]. Preparation of CDDP-loaded (*γ*-Fe_2_O_3_/PLGA)/CS particles was done by using the *γ*-Fe_2_O_3_/PLGA NPs with the higher DL values (i.e., ≈16%, see Section 3.3) (*n* = 3). NP production performance (PP, %) was calculated by using Equation (1):

(1) $$PP\;\left(\%\right)=\frac{\mathrm{amount}\;\mathrm{of}\;\mathrm{NPs}\;\mathrm{obtained}\;\left(\mathrm{mg}\right)}{\mathrm{summation}\;\mathrm{of}\;\mathrm{materials}\;\mathrm{used}\;\mathrm{in}\;\mathrm{the}\;\mathrm{preparation}\;\mathrm{of}\;\mathrm{these}\;\mathrm{NPs}\;\left(\mathrm{mg}\right)}\times 100$$

### 2.3. Characterization

Size and polydispersity index (PdI) of the NPs, and zeta potential (*ζ*) of the aqueous NP dispersions (≈0.1%, *w/v*) were characterized at room temperature and in triplicate (Zetasizer Nano-ZS, Malvern Instruments Ltd., Worcestershire, UK), by photon correlation spectroscopy (PCS) and electrokinetic determinations, respectively. The detection angle was 60°. Furthermore, annular bright field scanning transmission electron microscopy (ABF-STEM), high-angle annular dark field scanning transmission electron microscopy (HAADF-STEM), and high-resolution transmission electron microscopy (HRTEM) (Titan G2 60-300 FEI microscope, Thermo Fisher Scientific Inc., Waltham, MA, USA; accelerating voltage of 300 kV) facilitated the visualization the particles. Drops of the colloids (≈0.1%, *w/v*) were poured on formvar/carbon-coated copper microgrids and dried at room temperature. During the TEM determinations, elemental analysis was done [energy dispersive X-ray (EDX) spectrometer, Bruker Nano GmbH, Berlin, Germany]. Finally, short-term stability of an aqueous dispersion of (core/shell)/shell particles (1 mg/mL, pH ≈ 6) at 25.0 ± 0.5 °C was tested in triplicate. Evolution of size, PdI and *ζ* values of the NPs was measured as a function of time.

Interaction of the nanocomposites with human serum proteins for possible protein corona formation was analysed in vitro, following a previously detailed methodology [69,70]. To that aim, 70 µL of the magnetic colloid (9.5 mg/mL) were incubated during 40 min at 37.0 ± 0.5 °C in 5 mL of PBS (final concentration of 130 μg/mL of PBS) containing 0, 34 or 54 mg/mL of BSA. Size and PdI was then determined by PCS after magnetic removal of the NPs from the media (0.4 T permanent magnet) and redispersion in water (≈0.1%, *w/v*).

Electrokinetic determinations were done to define qualitatively the surface disposition of CS onto the (*γ*-Fe_2_O_3_/PLGA) NPs, given that the technique is most sensitive to small modifications on the surface of iron oxide NPs [22,71]. To that objective, the evolution of the *ζ* values as a function of the ionic strength (at a constant pH ≈ 6) was characterized. KNO_3_ was the electrolyte used to fix the ionic strengths [32,72,73], and the determinations were done at room temperature, after 24 h of contact under stirring (200 rpm, Boeco universal orbital shaker OS-10, Boeco, Hamburg, Germany) (*n* = 9). With this aim, chemical characterization of the (*γ*-Fe_2_O_3_/PLGA)/CS particles was also done by Fourier transform infrared (FTIR) spectrometry (FT/IR-6200 spectrometer, JASCO, Easton, MD, USA; resolution of 0.25 cm^−1^). Significant bands of these nanocomposites were identified by comparison with published data.

Given that the internal structure of iron oxide-based particles is considered to be a key factor defining their magnetism [22,74], determination of the mineralogical purity and crystallinity of the *γ*-Fe_2_O_3_ cores and the (core/shell)/shell particles was accomplished by X-ray diffractometry (Philips PW1710 diffractometer, Eindhoven, The Netherlands). With this aim, the Debye-Scherrer method was used (Cu-K*α* radiation of *λ* = 1.5405 Å).

Magnetic properties of the (*γ*-Fe_2_O_3_/PLGA)/CS particles were investigated at 25.0 ± 0.5 °C, under the effect of a magnetic field of 0 to 4000 kA/m (Manics DSM-8 vibrating magnetometer, Toulouse, France). As well, optical visualization of the magnetic responsiveness of the (core/shell)/shell NPs to a 400 mT permanent magnet, placed close to the glass vial containing the colloid (≈0.1%, *w/v*), was done. These are in-vitro experiments widely used to evaluate the magnetic responsiveness of a nanoparticulate system, as previously defined in the literature [22,32,75,76,77].

### 2.4. Hemocompatibility

One important aspect to consider when defining the clinical use of a nanoparticulate system is how the colloid interacts with blood, and the consequences, e.g., impact on erythrocytes, coagulation, and complement system. In this study, it was followed a previously described procedure appropriate to nanopharmaceutics [67,78,79]. Briefly, blood samples, taken from three healthy adults (22, 26, and 43 years old), were poured into flasks containing EDTA (before the haemolysis, and platelet activation experiments), or NaH_2_(C_3_H_5_O (COO)_3_) (before the complement system activation, and plasma clotting time assays). PBS was the negative control used in the experiments. The *γ*-Fe_2_O_3_ or the (*γ*-Fe_2_O_3_/PLGA)/CS particles were kept in contact with the samples to evaluate their interaction with blood. Validated UV spectrophotometric method was used in the assay which was done in triplicate.

### 2.5. In-Vitro Quantification of CDDP Loading

Evaluation of the amount of antitumour agent loaded to the *γ*-Fe_2_O_3_/PLGA and (*γ*-Fe_2_O_3_/PLGA)/CS particles was done in triplicate by UV spectrophotometric determinations of the CDDP molecules remaining in the supernatant after NP centrifugation (60 min and 11,000 rpm) (Centrifuge 5804, Eppendorf Ibérica S.L.U., Madrid, Spain). Considering that quantity was not loaded and the total amount of chemotherapeutic was used, the incorporation of CDDP to the NPs was determined. In these quantifications, contributions to the absorbance of sources other than variations in drug concentration, i.e., PVA, CH_3_COOH, and Kolliphor^®^ P-188, were taken into account by subtracting the absorbance of the supernatant produced under the same conditions but without CDDP.

These UV absorption measurements were performed at the maximum absorbance wavelength of this chemotherapeutic (301 nm) (Lambda™ 25 UV/Vis spectrophotometer, PerkinElmer Inc., Waltham, MA, USA). Good linearity was observed at this wavelength between absorbance and drug concentration data (*r* = 0.997), and the method was validated and verified for precision, accuracy, and linearity. CDDP incorporation to the magnetopolymeric NPs was calculated in terms of DL (%) (Equation (2)):

(2) $$DL\;\left(\%\right)=\frac{\mathrm{Entrapped}\;\mathrm{CDDP}\;\left(\mathrm{mg}\right)}{\mathrm{CDDP}-\mathrm{loaded}\;\mathrm{NPs}\;\left(\mathrm{mg}\right)}\times 100$$

### 2.6. In-Vitro Quantification of CDDP Release

The CDDP release experiments were based on the dialysis bag method (*n* = 3), and they were carried out using the (*γ*-Fe_2_O_3_/PLGA)/CS particles with the higher DLs (≈15%, see Section 3). The release media were either C_6_H_8_O_7_-Na_2_HPO_4_ or C_6_H_8_O_7_-NaOH buffers reproducing the pH of bloodstream (7.4 ± 0.1) or the acidic microenvironment in tumours (pH 5.0 ± 0.1) [80], respectively. These in-vitro drug release media were kept either at the normal human body-temperature (normothermia or euthermia, 37.0 ± 0.5 °C), or at the representative temperature of magnetic hyperthermia-triggered drug release experiments (45.0 ± 0.5 °C) [81,82,83]. The latter temperature is also the characteristic maximum hyperthermia temperature of magnetic colloids commonly used as magnetic hyperthermia agents for cancer treatment [83,84].

Before use, the dialysis bags (cut-off of 2000 Da, Spectrum^®^ Spectra/Por^®^ 6 dialysis membrane tubing, New Brunswick, NJ, USA) were soaked in water at room temperature for 12 h. Then, 2 mL of the dispersion of CDDP-loaded (*γ*-Fe_2_O_3_/PLGA)/CS NPs (containing 2.5 mg/mL of chemotherapy agent) were poured into the bag with the two ends fixed by clamps. The dialysis bags were placed in a glass beaker containing 0.1 L of release medium and stirred at 100 rpm. At prefixed time intervals, one mL of the medium was withdrawn for UV spectrophotometric analysis of the CDDP content (at 301 nm). An equal volume of the release media, kept also at 37.0 ± 0.5 or 45.0 ± 0.5 °C, was added after sampling to maintain the *sink* conditions. The same analytical procedure used to determine the DL (%) was used, and the in-vitro CDDP release was determined by using Equation (3):

(3) $$CDDP\;released\;\left(\%\right)=\frac{\mathrm{Amount}\;\mathrm{of}\;\mathrm{CDDP}\;\mathrm{released}\;\left(\mathrm{mg}\right)}{\mathrm{Amount}\;\mathrm{of}\;\mathrm{CDDP}\;\mathrm{loaded}\;\mathrm{to}\;\mathrm{the}\;\mathrm{NPs}\;\left(\mathrm{mg}\right)}\times 100$$

### 2.7. In-Vitro Cytotoxicity Assays

Blank (drug-unloaded) (*γ*-Fe_2_O_3_/PLGA)/CS NPs were tested in human colonic CCD-18 fibroblasts (Scientific Instrumentation Centre, University of Granada, Granada, Spain), and in human colon carcinoma T-84 cells (American Type Culture Collection, Manassas, VA, USA). According to the ISO-10993-5 standard [85], cell viability was tested (in triplicate) by the MTT proliferation assay, determining mitochondrial dehydrogenase activity. Detailed methodology has been described in the literature [67,79]. The cells were kept in contact with NP concentrations, ranging from 0.05 to 100 μg/mL, for 48 and 72 h at 37.0 ± 0.5 °C in a humidified atmosphere of 5% CO_2_ in air (MCO-19AIC(UV) CO_2_ incubator, Sanyo, Osaka, Japan).

Cytotoxicity of the CDDP-loaded (core/shell)/shell particles against human lung adenocarcinoma A-549 cells (American Type Culture Collection, Manassas, VA, USA) was evaluated by the MTT assay in comparison with the free chemotherapy agent (*n* = 3). The amount of these formulations in contact with the tumour cells ranged from 1 to 20 µg/mL equivalent CDDP concentrations (NP concentrations from ≈ 6.5 to 130 μg/mL).

Cells without treatment were used as control to calculate the relative cell viability (RCV, %) (Equation (4)).

(4) $$RCV\;\left(\%\right)=\frac{\mathrm{Optical}\;\mathrm{density}\;\mathrm{of}\;\mathrm{treated}\;\mathrm{cells}}{\mathrm{Optical}\;\mathrm{density}\;\mathrm{of}\;\mathrm{control}\;\left(\mathrm{untreated}\right)\;cells}\times 100$$

The half maximal inhibitory concentration (IC_50_) values were estimated by a non-linear regression analysis (GraphPad Prism 9.1.0, GraphPad Software Inc., La Jolla, CA, USA) [86,87].

### 2.8. Statistical Analysis

The IBM^®^ SPSS^®^ Statistics 26.0 software package (IBM Corporation, New York, NY, USA) was used to that aim. Student’s t-test was done to compare results considering 95% confidence interval. The experimental data were expressed as mean value ± SD, and the differences were considered to be statistically significant at *p* < 0.05, or highly significant at *p* < 0.01.

## 3. Results and Discussion

On the basis of the experimental data collected in this Section, it is discussed how the (*γ*-Fe_2_O_3_/PLGA)/CS (core/shell)/shell NPs are characterized by an appropriate size and blood compatibility for intravenous administration, also probably by stealth properties; and, more interestingly, by adequate magnetic-, pH-, and heat (hyperthermia)-sensitivities. All these characteristics could make the nanocomposites an interesting nanocarrier for CDDP: site-specific delivery of the antitumour agent could be considerably optimized, reaching therapeutic levels inside the malignant cells. Of course, defining the real possibilities of these CDDP-loaded nanostructures against lung cancer will only be possible if additional in-vitro tests and, more significantly, experiments in tumour-bearing are done.

### 3.1. Characterization of the Nanoparticulate Systems

Mean diameter (and PdI) of *γ*-Fe_2_O_3_ nuclei, pure CS particles, and (*γ*-Fe_2_O_3_/PLGA) NPs were: 10.2 ± 2.1, 364.1 ± 15.3, and 291.2 ± 1.8 nm, respectively. The methodology of preparation of the (*γ*-Fe_2_O_3_/PLGA)/CS NPs reported adequate sizes (332.1 ± 82.1 nm), and PPs (≈50%). These diameters are compatible with parenteral administration and could favour NP accumulation at the cancer site (gaps of up to ≈ 600 nm between endothelial cells of the vasculature) [22,88]. Some studies have reported that particles up to 400 nm may extravasate into the tumor tissue, taking advantage of the EPR effect [89,90,91]. For instance, CDDP-loaded particles with sizes from 300 to 500 nm have been described to reach the cancer site, inhibiting tumour growth after intravenous administration [60,92]. In addition, early plasma clearance could be minimized thanks to the stealth property that CS may provide to the (core/shell)/shell particles, and to their positive surface electrical charge (*ζ* value: 23.6 ± 0.3 mV) [45,93]. These positive *ζ* values may further promote NP internalization by the (negatively charged) cancer cell [49,94].

Regarding the short-term evaluation of the stability of these nanocomposites, data in Table 1 illustrates the absence of relevant changes in the size (and PdI) and surface electrical charge of the NPs. Therefore, it could be postulated that no particle aggregation will occur after one month of storage at room temperature in water. The PDI values were ≤0.5 during the study, similarly to what has been previously described for PLGA-based NPs surface functionalized with CS [95,96,97]. Such PDI data could be considered acceptable and characteristic of relatively homogeneous NP dispersions [98,99].

No significant effect on size was observed when the (*γ*-Fe_2_O_3_/PLGA)/CS NPs were kept in contact with BSA (34, and 54 mg/mL concentrations in PBS), being the particle diameter and PdI values ≈ 340 nm and ≈ 0.335, respectively. That negligible effect could be attributed to the hydrophilic nature of the CS coating which could minimize protein corona formation and particle aggregation [46,47].

**Figure 2:**
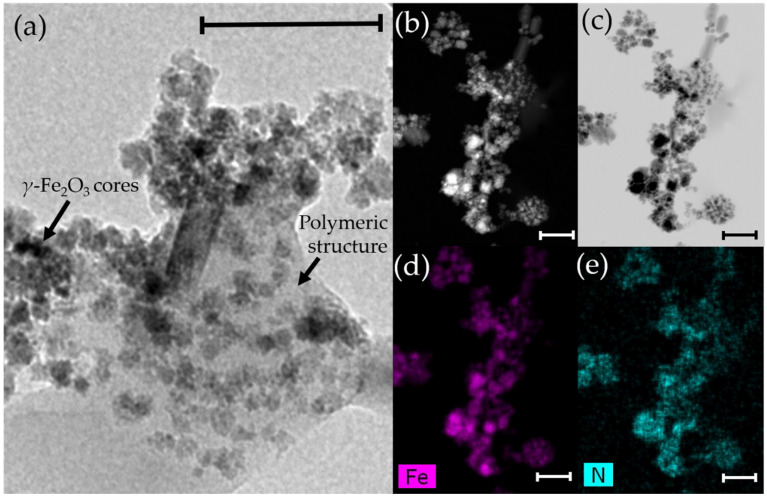
(**a**) HRTEM, (**b**) HAADF-STEM, and (**c**) ABF-STEM images of the (*γ*-Fe_2_O_3_/PLGA)/CS nanocomposites; EDX mapping analysis of the (**d**) Fe and (**e**) N elements of the sample in (**b**,**c**). Bar lengths: 100 nm. The arrows in (**a**) mark the *γ*-Fe_2_O_3_ cores inside the polymeric structure.

**Figure 3:**
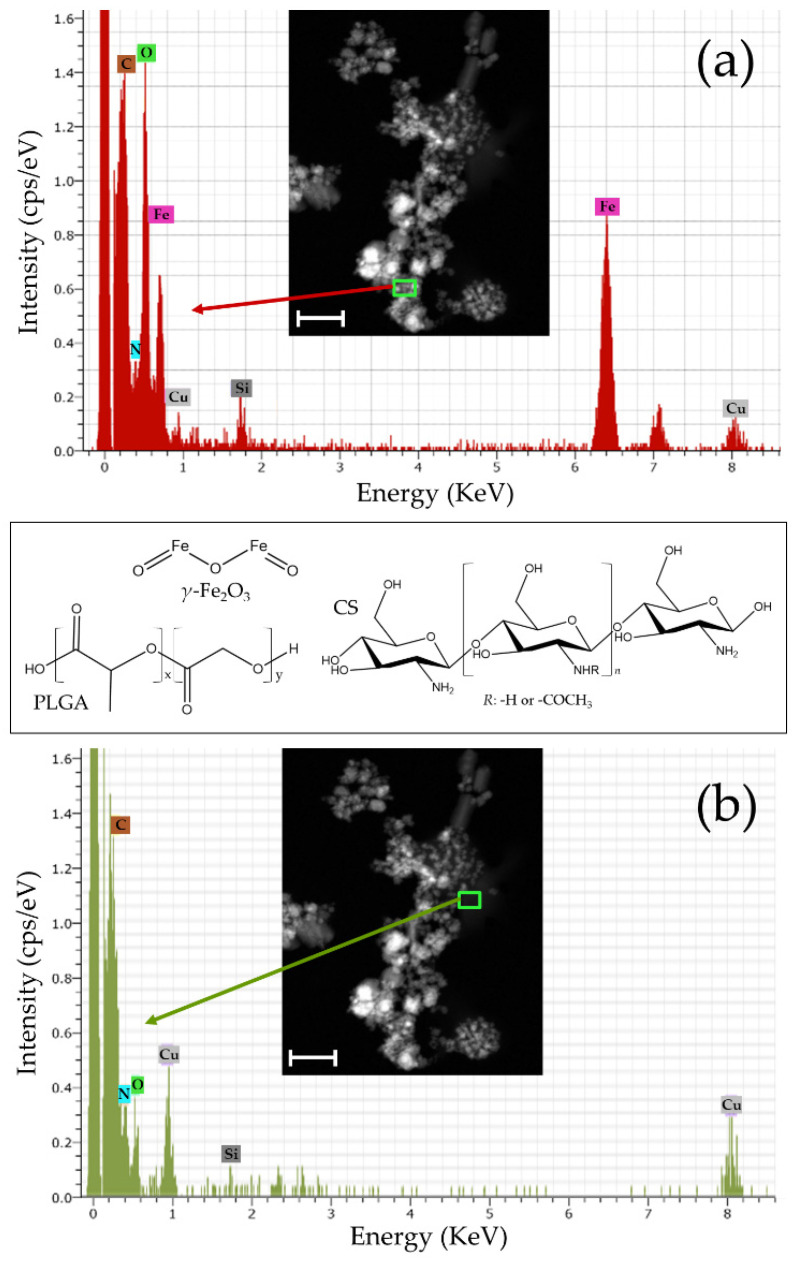
EDX spectra of: (**a**) the centre and (**b**) flank of the (core/shell)/shell NPs. Insets: HAADF-STEM images of these nanocomposites (bar lengths: 100 nm), and chemical structures of *γ*-Fe_2_O_3,_ PLGA and CS.

HRTEM (Figure 2a), HAADF-STEM (Figure 2b), and ABF-STEM (Figure 2c) photographs demonstrated the satisfactory embedment of the *γ*-Fe_2_O_3_ nuclei into the polymeric nanostructure. Particle aggregation observed in Figure 2 could be the consequence of the preparation of the samples by drying for the EM visualizations. This is a phenomenon previously described in CS-based magnetic nanocomposites [100,101] that determined the impossibility of identifying the shape of a single NP. On the other hand, the homogeneous distribution of these iron oxide nanocores within the particle matrix is visible in the EDX Fe element mapping of the (*γ*-Fe_2_O_3_/PLGA)/CS NPs (Figure 2d). Uniform coverage of the *γ*-Fe_2_O_3_/PLGA particles by the CS shell can be postulated from the EDX N element mapping of these (core/shell)/shell NPs (Figure 2e). Additionally, EDX analyses confirmed the presence of the Fe, C, N, and O elements for the nanocomposites (Figure 3). The Fe element arose from the *γ*-Fe_2_O_3_ cores and was only detected when the EDX spectrum was taken from the centre of the (core/shell)/shell particles (Figure 3a). On the opposite, the N element (from the CS shell) was identified in any EDX spectra taken of the NPs (Figure 3a,b), thus confirming the homogeneous disposition of CS onto the core/shell nanostructure. These observations established the formation of the (*γ*-Fe_2_O_3_/PLGA)/CS (core/shell)/shell nanostructure. Finally, the existence of the Cu and Si elements in the EDX analysis could arise from the use of copper-based grids [32] and the production of a secondary fluorescence by the fluorescence detector [102].

**Figure 4:**
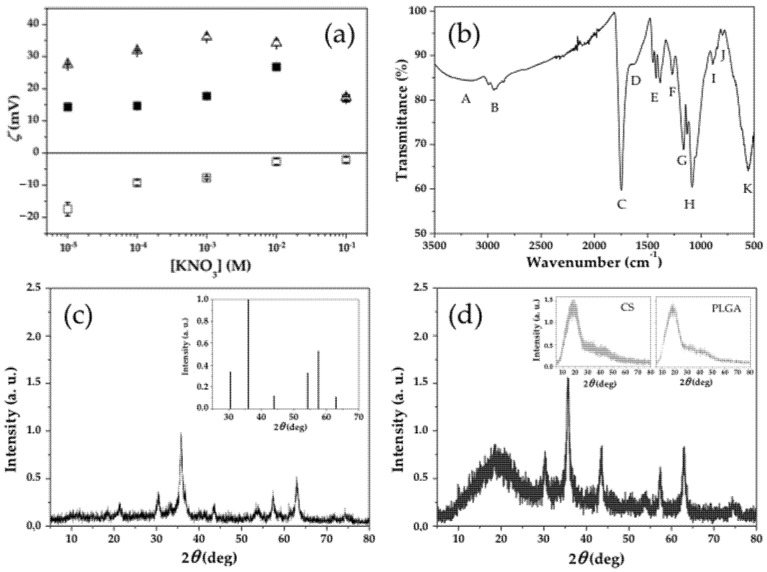
(**a**) Zeta potential (*ζ*, mV) of (□) *γ*-Fe_2_O_3_/PLGA NPs, (Δ) CS NPs, and (■) (*γ*-Fe_2_O_3_/PLGA)/CS NPs as a function of the molar concentration of KNO_3_, at pH ≈ 6. Data is presented as mean value ± SD (*n* = 9). (**b**) Infrared spectra of the (core/shell)/shell NPs. (**c**) X-ray diffractogram of the *γ*-Fe_2_O_3_ particles. Inset: American Society for Testing and Materials (ASTM) pattern for *γ*-Fe_2_O_3_. (**d**) X-ray diffractogram of the (*γ*-Fe_2_O_3_/PLGA)/CS NPs. Inset: X-ray diffractograms of the CS particles, and PLGA particles. The intensity is expressed in arbitrary units (a. u.).

Electrophoretic characterization of the colloids was then done to define qualitatively the surface disposition of CS onto the (*γ*-Fe_2_O_3_/PLGA) (core/shell) particles. Concretely, it was determined the surface electrical charge (*ζ* values) of the *γ*-Fe_2_O_3_/PLGA, CS, and (*γ*-Fe_2_O_3_/PLGA)/CS NPs at predetermined ionic strengths (KNO_3_ molar concentrations) and pH ≈ 6 (the natural pH of the colloids). Similarities between the *ζ* values of the CS and (*γ*-Fe_2_O_3_/PLGA)/CS particles, and how different they were from those of the *γ*-Fe_2_O_3_/PLGA NPs are plotted in Figure 4a. The negative surface charge of the *γ*-Fe_2_O_3_/PLGA particles may come from ionized weak acid groups, probably carboxylic-end groups of PLGA [103,104]. On the opposite, the positive *ζ* values of pure CS and (*γ*-Fe_2_O_3_/PLGA)/CS NPs could come from the residual amino groups existing in the chemical structure of CS [42,105]. Finally, the decrease in the absolute values of *ζ* described by the NPs at the larger ionic strengths should be the consequence of the classical double-layer compression mechanism [106,107]. It can be observed in Figure 4a how the *ζ*–ionic strength trend of the (*γ*-Fe_2_O_3_/PLGA)/CS NPs was dominated by the CS shell, probably being that the consequence of the effective CS coating of the (*γ*-Fe_2_O_3_/PLGA) particles. Finally, if the electrophoretic data in Figure 4a is considered, a mechanism could be postulated to explain the generation of the (core/shell)/shell NPs: accumulation of CS onto the *γ*-Fe_2_O_3_/PLGA surface may occur thanks to attractive interactions between the negatively charged core/shell particles and the positively charged CS matrix [52,108].

The infrared spectra of the (*γ*-Fe_2_O_3_/PLGA)/CS NPs is represented in Figure 4b. All the characteristics bands of the polymers (CS and PLGA) were present in the spectrum of these magnetopolymeric particles, hence demonstrating that the shell observed in Figure 2 corresponded well to the PLGA and CS shells onto the *γ*-Fe_2_O_3_ nuclei. Chemical groups identified in the spectra were: (A) overlapped stretching vibrations from N–H and O–H bonds (at ≈3400 cm^−1^) [109,110,111]; (B) C–H bond stretching vibration of −CH, −CH_2_, and −CH_3_ groups (at ≈2850 cm^−1^) [109,110,112]; (C) C = O bond stretching vibration of a carboxylic acid (at ≈1750 cm^−1^), probably from the PLGA shell [111,113,114]; (D) C = O bond stretching vibration of an amide group, presumably from the CS coating (≈1630 cm^−1^) [110,112,115]; (E) asymmetric CH_2_ bending vibration (at ≈1450 and ≈1380 cm^−1^) [110,112,113], and O–H bending vibration, probably from the carboxylic group in the PLGA shell (at ≈1420 cm^−1^) [104]; (F) C–O bond stretching vibrations from a −OH group (at ≈1280 cm^−1^) [116]; (G) C–O bond stretching vibration from the carboxylic group in PLGA (at ≈1160 cm^−1^) [104,116]; (H) C–O–C bond stretching vibration from PLGA (at ≈1130 and ≈1080 cm^−1^) [97,104,112]; (I) medium band characteristic of alkanes (at ≈890 cm^−1^) [117]; (J) CH rocking vibration characteristic of –CH long chains (at ≈800 cm^−1^) [104,117]; and, (K) Fe–O bond vibration from pure iron oxide NPs (at ≈560 cm^−1^) [104,118,119].

The X-ray diffraction patterns of *γ*-Fe_2_O_3_, and (*γ*-Fe_2_O_3_/PLGA)/CS NPs are plotted in Figure 4c,d, respectively. They coincided with the American Society for Testing and Materials (ASTM) pattern of *γ*-Fe_2_O_3_ (inset of Figure 4c) (ASTM No. 24-81). The experimental data suggested that, after complete inclusion into the PLGA and CS shells, the *γ*-Fe_2_O_3_ nuclei maintained a high crystallinity and mineralogical purity. These are properties beneath an appropriate magnetic responsiveness and a superparamagnetic behaviour [22]. 2*θ* values of the *γ*-Fe_2_O_3_ nanocores existing into the (*γ*-Fe_2_O_3_/PLGA)/CS nanostructure were 29.89°, 35.17°, 43.43°, 53.92°, 56.87°, and 63.12°, and they may be assigned to the (2 2 0), (3 1 1), (4 0 0), (4 2 2), (5 1 1), and (4 4 0) planes of these iron oxides, respectively [120,121,122]. In addition, broad diffraction peaks were observed for CS and PLGA particles at 2*θ* values ≈ 19.90° and ≈18.36°, respectively (insets to Figure 4d). The former peak would be indexed to the (1 1 0) plane that is characteristic of crystalline chitin [123,124,125], while the diffraction peak observed for PLGA NPs could have come from the amorphous phase of PLGA [126,127,128]. These broad peaks were integrated in the X-ray diffractogram of the (*γ*-Fe_2_O_3_/PLGA)/CS NPs (2*θ* value ≈ 18.57°), probably a confirmation of the efficiency of the preparation procedure in generating that (core/shell)/shell nanostructure.

**Figure 5:**
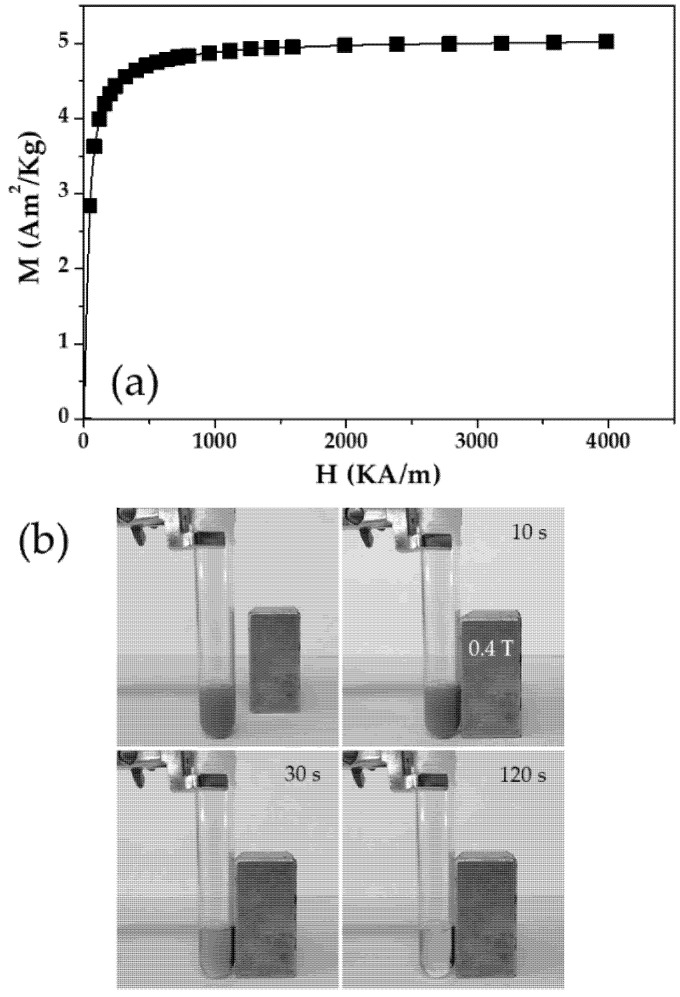
(**a**) First magnetization curve of the (core/shell)/shell NPs. (**b**) Visual observation of the colloid (0.1%, *w/v*) under the influence of 0.4 T permanent magnet, located close to the right lateral flat surface of the glass vial.

The first magnetization curve of the (*γ*-Fe_2_O_3_/PLGA)/CS NPs, shown in Figure 5a, characterized the appropriate magnetic responsiveness of this magnetic colloid. Initial susceptibility and saturation magnetization values of the (core/shell)/shell particles were (0.077 ± 0.003) × 10^−3^ m^3^/Kg and 5.03 ± 0.37 Am^2^/Kg, respectively. That adequate magnetic responsive behaviour was further qualitatively confirmed by visual observation of the colloid under exposure to a permanent magnet (Figure 5b): complete magnetic attraction of the NPs toward the 400 mT magnet occurred in 120 s. However, in-vivo experiments should be performed to define if this magnetic responsiveness could favour the accumulation of the (*γ*-Fe_2_O_3_/PLGA)/CS particles at a targeted site.

### 3.2. Cytotoxicity and Blood Compatibility

**Figure 6:**
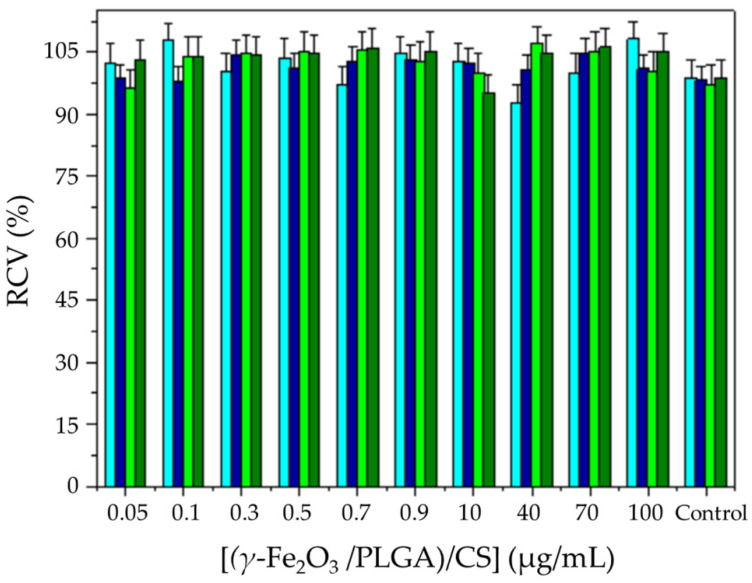
In-vitro cytotoxicity (RCV, %) of the (*γ*-Fe_2_O_3_/PLGA)/CS NPs in CCD-18 cells at 48 h (blue columns) and 72 h (green columns), and in T-84 cells at 48 h (dark blue columns) and 72 h (dark green columns). These cell lines were kept in contact with NP concentrations, ranging from 0.05 to 100 μg/mL.

Data from the evaluation of the cytotoxicity of (*γ*-Fe_2_O_3_/PLGA)/CS NPs in normal CCD-18 and tumour T-84 cells are represented in Figure 6, which illustrates how viability of both cell lines was not altered by these (core/shell)/shell particles, even when the NP concentration was increased from 0.05 to 100 μg/mL. According to ISO-10993-5 [85], the RCV (%) values plotted in Figure 6 could be considered to be non-toxic. Additionally, growth of cells kept in contact with non-cytotoxic blank (drug-unloaded) NPs has been described to be not hindered, even at high concentrations [129,130,131]; as a result, proliferation can continue under in-vitro conditions. On the other hand, experimental results from the ex-vivo hemocompatibility assays of *γ*-Fe_2_O_3_ and (*γ*-Fe_2_O_3_/PLGA)/CS particles demonstrated a negligible effect on haemolysis, platelet activation, complement system activation, and plasma clotting time was observed (Table 2). Therefore, taking into account the results from Figure 6 and Table 2, it could be postulated that the (*γ*-Fe_2_O_3_/PLGA)/CS NPs are characterized by an adequate biocompatibility and safety for drug delivery purposes, being suitable for parenteral administration.

### 3.3. CDDP Loading

The conditions fixed to prepare the CDDP-loaded *γ*-Fe_2_O_3_/PLGA NPs tried to minimize the escape of this hydrophilic drug [132] from mechanical trapping into the hydrophobic PLGA matrix [104]. It has been previously determined the very rapid precipitation of the polymer matrix, just upon contacting the H_2_O phase, when PLGA NPs are prepared by the nanoprecipitation solvent evaporation technique [103,133]. As a consequence, mechanical trapping of the drug inside that polymer network would be facilitated [134]. Complementarily, stabilizing agents, e.g., poloxamers, PVA, may induce the opening of the polymer chains to create a space within the PLGA matrix where the drug could be incorporated [133,135]. Furthermore, CDDP incorporation to the PLGA matrix could be the consequence of electrostatic attractions between drug molecules, positively charged when the –NH group is protonated, and the negatively charged polymer (≈−10 mV at natural pH 6). Electrostatic repulsions between the positively charged CDDP molecules and the positively charged *γ*-Fe_2_O_3_ nuclei (≈+20 mV at natural pH 6) of these nanostructures may prevent drug adsorption onto these iron oxides.

CDDP loading values to the *γ*-Fe_2_O_3_/PLGA, and (*γ*-Fe_2_O_3_/PLGA)/CS nanocomposites are compiled in Table 3. As expected, drug concentration positively influenced CDDP absorption into the core/shell nanostructure, while no relevant modification of the DL (%) values was observed when the *γ*-Fe_2_O_3_/PLGA particles were surface coated with CS. In addition, CDDP incorporation to the (*γ*-Fe_2_O_3_/PLGA)/CS particles reported greater DL values (≈15%) than those previously ascribed to non-magnetic PLGA/CS-based particles (DL ≈ 9%) [49]. Finally, particle diameter and surface electrical charge of the (core/shell)/shell particles did not vary when loaded with the chemotherapy agent: ≈325 nm and ≈+23 mV, respectively. The great similarity between the *ζ* values of the non-loaded and the CDDP-loaded (*γ*-Fe_2_O_3_/PLGA)/CS particles may suggest an efficient absorption of the CDDP molecules into the nanocomposites.

### 3.4. CDDP Release

**Figure 7:**
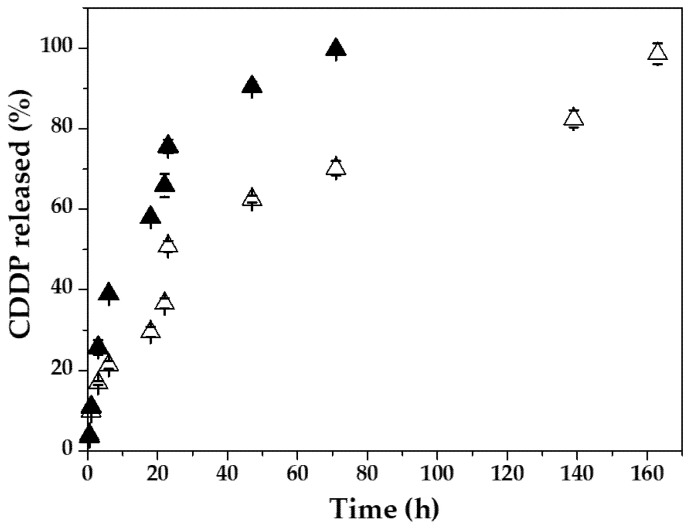
In-vitro release of CDDP (%) from the (*γ*-Fe_2_O_3_/PLGA)/CS (core/shell)/shell NPs as a function of the incubation time (h) at 37.0 ± 0.5 °C, and pH 7.4 ± 0.1 (Δ) or pH 5.0 ± 0.1 (▲). Data is presented as mean value ± SD (*n* = 3).

The pH-responsive CDDP release from the (*γ*-Fe_2_O_3_/PLGA)/CS NPs was evaluated at 37.0 ± 0.5 °C by using release media reproducing either the pH ≈ 7.4 of bloodstream or the acidic microenvironment in tumours (pH ≈ 5) (Figure 7). A biphasic drug release profile was identified, which is characteristic of PLGA-based [58,67] and CS-based [42,60] particles, and has been previously proposed for PLGA/CS (core/shell) nanostructures loaded with Paclitaxel [49], and Itraconazole [136]. In detail, the process started with an early-time burst drug release, taking place in ≈6 h (up to ≈21% at pH 7.4, and ≈39% at pH 5.0). The remaining chemotherapeutic was then released slowly during around the next 154 h at pH 7.4, and 66 h at pH 5.0. Such a biphasic CDDP release profile could be attributed to drug diffusion through the PLGA/CS architecture in the initial stage and to degradation/erosion of these polymer matrices during the final phase of drug release [50,67,137], and may further suggest that the major proportion of CDDP molecules was entrapped efficiently into the PLGA shell. Significantly, the pH-responsive CDDP release behaviour was identified in Figure 7: ≈1.4-fold faster drug release at pH 5.0 compared to pH 7.4 (*p* < 0.05). In comparison with blood, the rapid drug release at the acidic intratumoural pH may be the consequence of the higher solubility of CS at lower pH values [42,43] and the accelerated degradation of PLGA by increased hydrolysis of the backbone ester linkages in its chemical structure [36,37]. The existence of hydrophilic CS shell onto the PLGA matrix could enhance the interaction of the NP with the acidic aqueous medium, thus facilitating a faster degradation of PLGA by hydrolysis and, consequently, the more rapid CDDP release compared to what occurred at the pH 7.4 of bloodstream [36].

**Figure 8:**
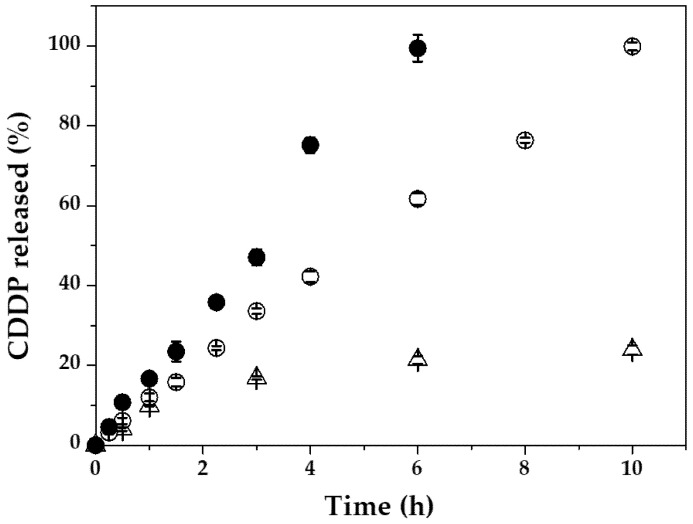
In-vitro release of CDDP (%) from the (*γ*-Fe_2_O_3_/PLGA)/CS (core/shell)/shell NPs as a function of the incubation time (h) at pH 7.4 ± 0.1 and 37.0 ± 0.5 °C (Δ), pH 7.4 ± 0.1 and 45.0 ± 0.5 °C (○), and pH 5.0 ± 0.1 and 45.0 ± 0.5 °C (●).

The in-vitro CDDP release was considerably augmented when the NPs were kept at the temperature commonly established in magnetic hyperthermia to trigger drug release (45.0 ± 0.5 °C) [81,82,83,138]. At this temperature, CDDP release was a very rapid process (Figure 8), decelerated when the (core/shell)/shell particles were kept at the physiological pH of 7.4. Concretely, drug release was completed in ≈10 h at pH 7.4, and in ≈6 h at pH 5.0. The fast CDDP release from the (*γ*-Fe_2_O_3_/PLGA)/CS NPs could be resulting from both the enhanced permeability toward water and solutes displayed by the PLGA shell at temperatures over the *T*_g_ [39] and the heat-mediated degradation of PLGA [40].

Taking into account the data in the Figure 7 and Figure 8, it could be postulated that the (*γ*-Fe_2_O_3_/PLGA)/CS particles can display a pH- and heat-responsive CDDP release behaviour, generating a ≈4.7-fold faster drug release at pH 5.0 and 45 °C compared to physiological conditions (pH 7.4 and 37 °C) (*p* < 0.05). This value was estimated at the 6 h time point: the moment when all the drug was released at pH 5.0 and 45 °C, while only ≈21% of CDDP was released at pH 7.4 and 37 °C. Finally, and taking into account the data plotted in the Figure 5 and Figure 7, the CS-decorated particles could be considered magnetic-, pH- and heat (hyperthermia)-responsive nanostructures probably facilitating in vivo the selective accumulation of CDDP molecules into the tumour interstitium or even intracellularly (inside the lysosomes of malignant cells after cellular uptake) [139,140,141].

### 3.5. Cytotoxicity against Human Lung Adenocarcinoma A-549 Cells

**Figure 9:**
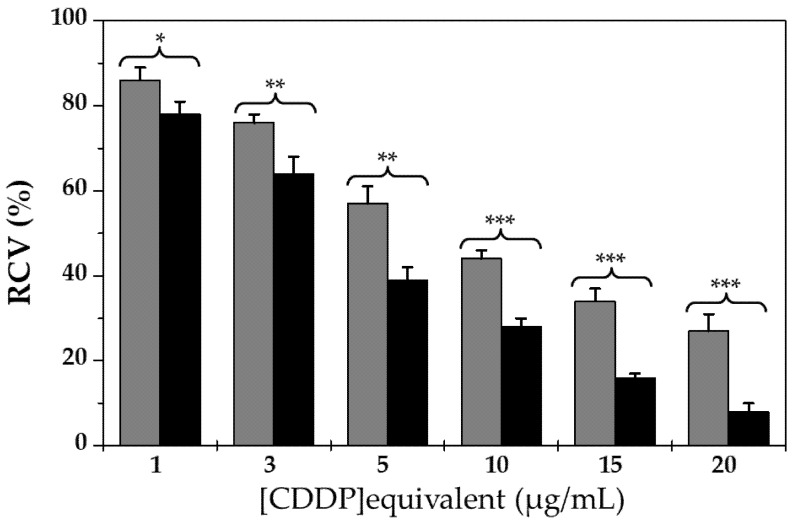
In-vitro cytotoxicity (RCV, %) of CDDP-loaded (*γ*-Fe_2_O_3_/PLGA)/CS NPs (black column) and free CDDP (light grey column) in human lung adenocarcinoma A-549 cells, after 72 h of exposure to a wide range of NP concentrations (up to 20 μg/mL equivalent CDDP concentration). The statistical Student’s t-test, considering 95% confidence interval, was significant (* *p* < 0.05) or very significant (** *p* < 0.01, *** *p* < 0.001), compared with the free CDDP-treated group.

Figure 9 illustrates the dose-dependent inhibition of cancer cell growth displayed by the CDDP formulations. At 3 to 20 μg/mL equivalent CDDP concentrations (NP concentrations from ≈20 to 130 μg/mL)), it was observed a very significant enhancement of the antitumour activity of the chemotherapeutic when being loaded to the (*γ*-Fe_2_O_3_/PLGA)/CS particles (*p* < 0.01). They were the nanoformulations that could be considered with cytotoxic potential, according to the ISO-10993-5 standard (RCV values < 70%) [85]. Furthermore, the half maximal inhibitory concentration (IC_50_) of the CDDP-loaded (core/shell)/shell NPs (4.57 ± 0.33 μg/mL) was ≈ 1.6-fold less than that of this platinum-based anticancer drug (7.48 ± 0.37 μg/mL) (*p* < 0.05). This greater in-vitro cytotoxic activity agrees with preceding research in which CDDP loading to nanoparticulate systems is postulated to enable cellular uptake in malignant tissues [142,143].

Complementary in-vitro tests will help in providing clear evidence of the successful delivery of CDDP to tumour cells, and the real possibilities of these (core/shell)/shell NPs against lung cancer. For instance, the apoptosis assay involving annexin V/propidium iodide staining, the crystal violet staining experiment determining viability of cultured cells, and colony forming and cell migration assays to characterize the proliferative competence of the cancer cells on exposure to the CDDP-loaded NPs. Experiments in tumour-bearing mice will be also needed to clearly define the anticancer effect of the CDDP-loaded nanocomposites.

## 4. Conclusions

A reproducible procedure has been developed to prepare (*γ*-Fe_2_O_3_/PLGA)/CS (core/shell)/shell NPs loaded with the chemotherapeutic CDDP (≈330 nm in size, PP ≈ 50%, DL ≈ 15%). That nanostructure was reasonably characterized by EM, EDX, FTIR, and electrophoretic analyses. Short-term stability of the magnetic colloid was defined at room temperature. The high crystallinity and mineralogical purity of the iron oxide nuclei in the polymer matrices was proved by X-ray diffraction analysis, being that these properties are possibly underneath the appropriate magnetism of the (*γ*-Fe_2_O_3_/PLGA)/CS particles (demonstrated in vitro). Data from the cytotoxicity assays and ex-vivo hemocompatibility tests may suggest the in-vivo compatibility and safety of the (core/shell)/shell NPs, being that they are suitable for parenteral administration (and drug delivery). Furthermore, CDDP incorporation to this nanostructure generated a dual pH- and heat (hyperthermia)-responsive drug release behaviour (≈4.7-fold faster CDDP release at pH 5.0 and 45 °C compared to pH 7.4 and 37 °C). To end, the CDDP-loaded (*γ*-Fe_2_O_3_/PLGA)/CS particles demonstrated a better cytotoxicity than the free CDDP molecules against human lung adenocarcinoma A-549 cells (IC_50_ ≈ 1.6-fold less than that of the chemotherapeutic) in absence of an applied magnetic field. Altogether, these biocompatible and tri-stimuli responsive (*γ*-Fe_2_O_3_/PLGA)/CS particles may become a contender in the lung cancer arena. Additional in-vitro and in-vivo experiments will contribute to the perfect definition of the therapeutic effectiveness of this nanostructure in cancer chemotherapy.

## Figures and Tables

**Table 1:** Particle diameter (nm), PdI, and *ζ* values (mV) of the (core/shell)/shell NPs as a function of time (days). Data are expressed as means ± SDs of triplicate experiments.

Time	Day 0	Day 1	Day 7	Day 14	Day 30
**Size (nm)**	332.1 ± 82.1	329.9 ± 168.3	333.8 ± 105.3	321.5 ± 97.8	334.9 ± 71.4
**PdI**	0.375 ± 0.006	0.422 ± 0.075	0.403 ± 0.036	0.289 ± 0.052	0.412 ± 0.077
**ζ (mV)**	23.6 ± 0.3	17.3 ± 0.7	23.3 ± 0.5	14.7 ± 1.8	26.3 ± 0.4

**Table 2:** Effect of the γ-Fe_2_O_3_, and (γ-Fe_2_O_3_/PLGA)/CS NPs on haemolysis (%), complement activation (C3a release: C3a desArg, ng/mL), platelet activation (sP-selectin release, ng/mL), and plasma recalcification time (T_1/2 max_, min). Data is indicated as means ± SDs (*n* = 3).

	*γ*-Fe_2_O_3_ NPs	(*γ*-Fe_2_O_3_/PLGA)/CS NPs	Control (PBS Solution)
**Haemolysis (%)**	1.6 ± 0.1	2.5 ± 0.4	0
**C3a desArg (ng/mL)**	296 ± 3	309 ± 8	290 ± 9
**sP-selectin release (ng/mL)**	105 ± 6	117 ± 5	99 ± 6
**T_1/2 max_ (min)**	14.1 ± 0.7	13.8 ± 1.2	12.2 ± 0.9

**Table 3:** Loading of CDDP (DL, %) to the *γ*-Fe_2_O_3_/PLGA, and (*γ*-Fe_2_O_3_/PLGA)/CS particles.

Nanoparticulate System	[CDDP] (μg/mL)	DL (%)
*γ*-Fe_2_O_3_/PLGA	3	0.021 ± 0.003
	15	0.212 ± 0.079
	30	0.995 ± 0.163
	150	5.945 ± 0.364
	300	16.057 ± 2.359
(*γ*-Fe_2_O_3_/PLGA)/CS	300	14.974 ± 3.025

## Data Availability

The experimental data have been provided within the manuscript.
